# Supplementary material for: Mucoricin binding to β-glucan sites on germinating Mucorales spores disrupts neutrophil swarming to promote pathogenicity
Source: bioRxiv. 2025 Oct 29:2025.10.28.685056. Preprint. [Version 1] doi: 10.1101/2025.10.28.685056 (PMC12636483; doi:10.1101/2025.10.28.685056)

Supplementary Figure 1

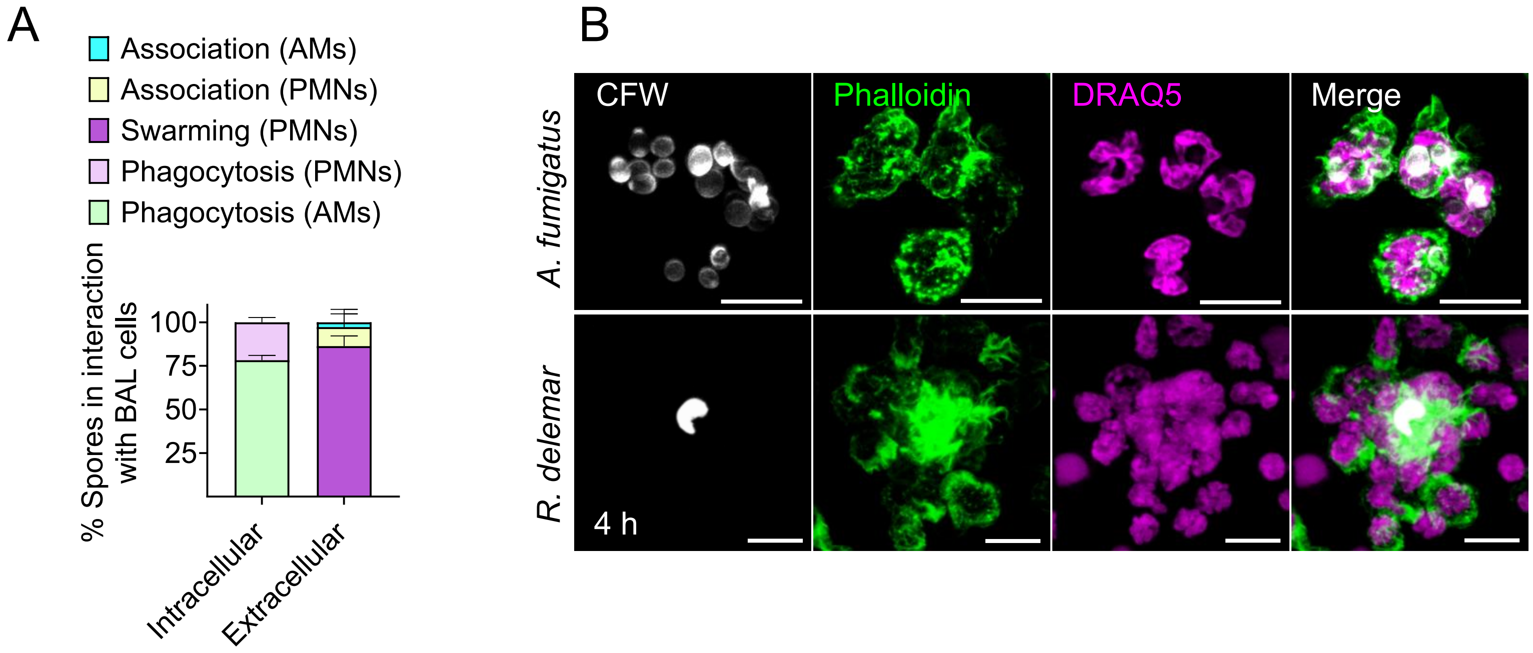

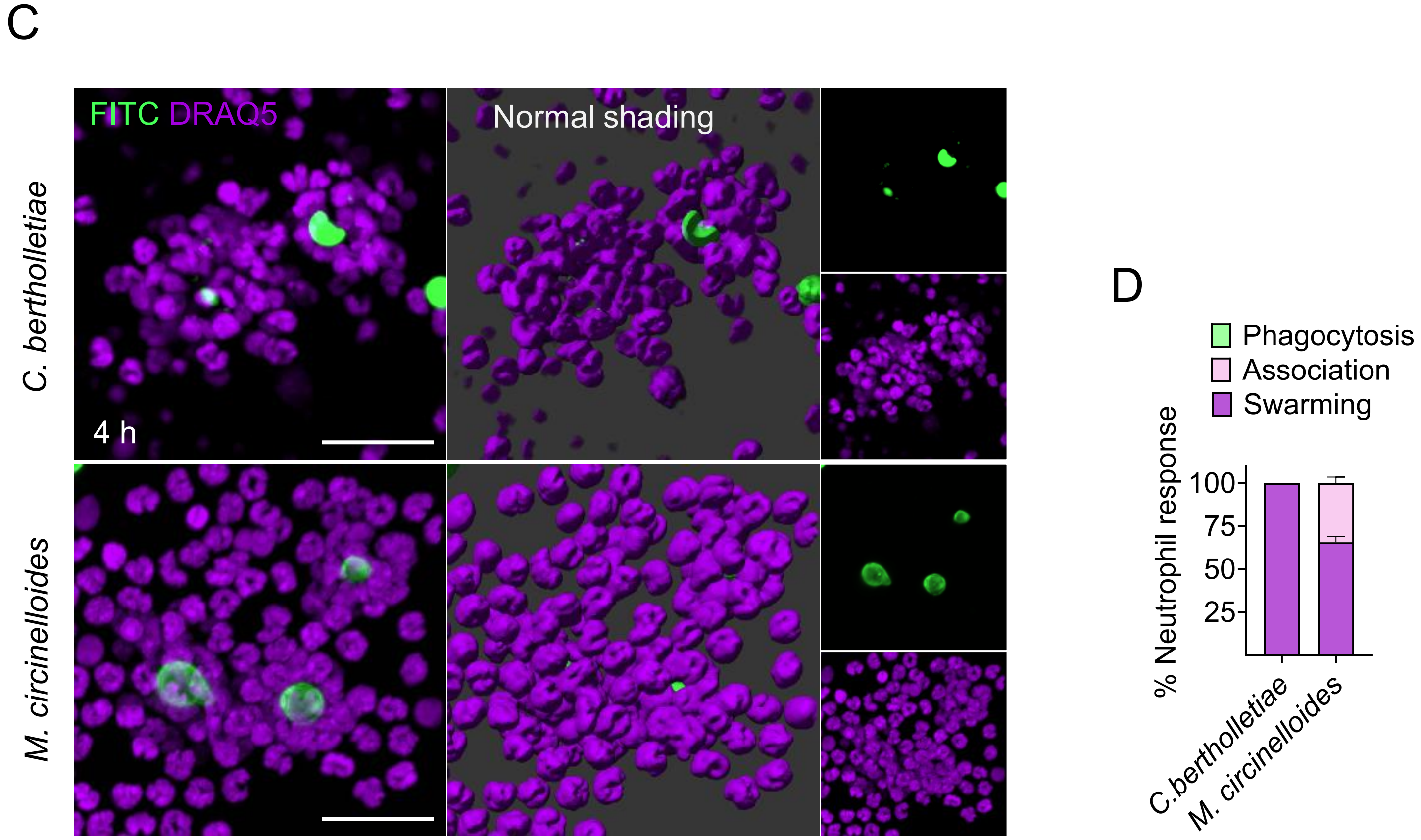

Supplementary Figure 2

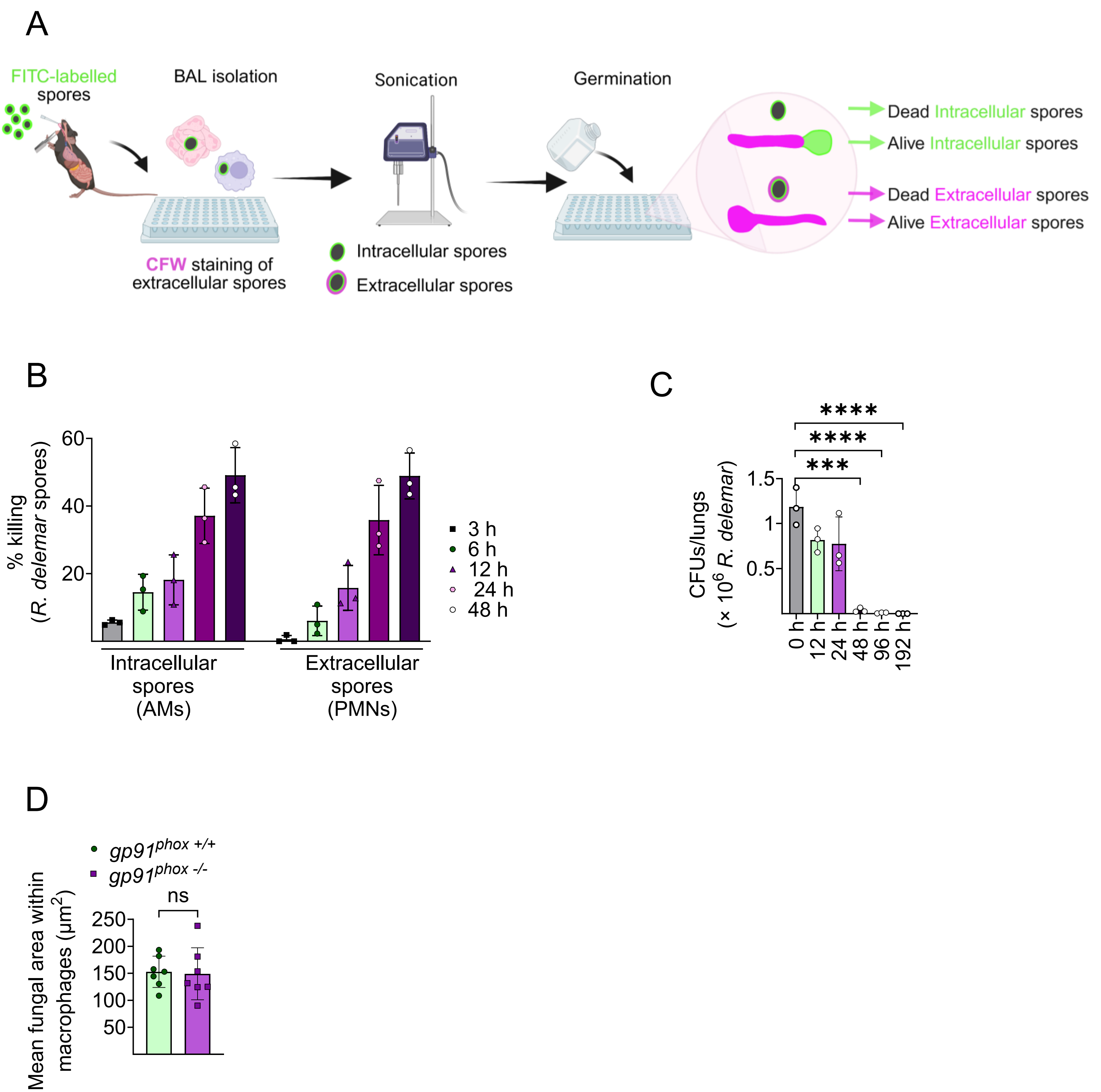

Supplementary Figure 3

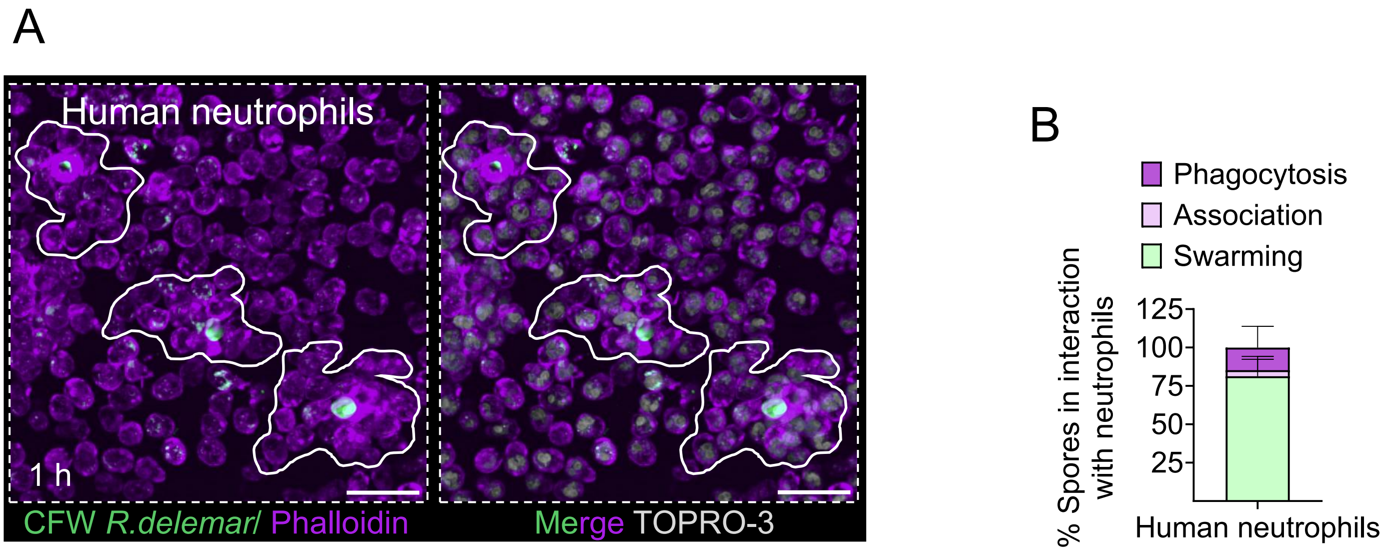

Supplementary Figure 4

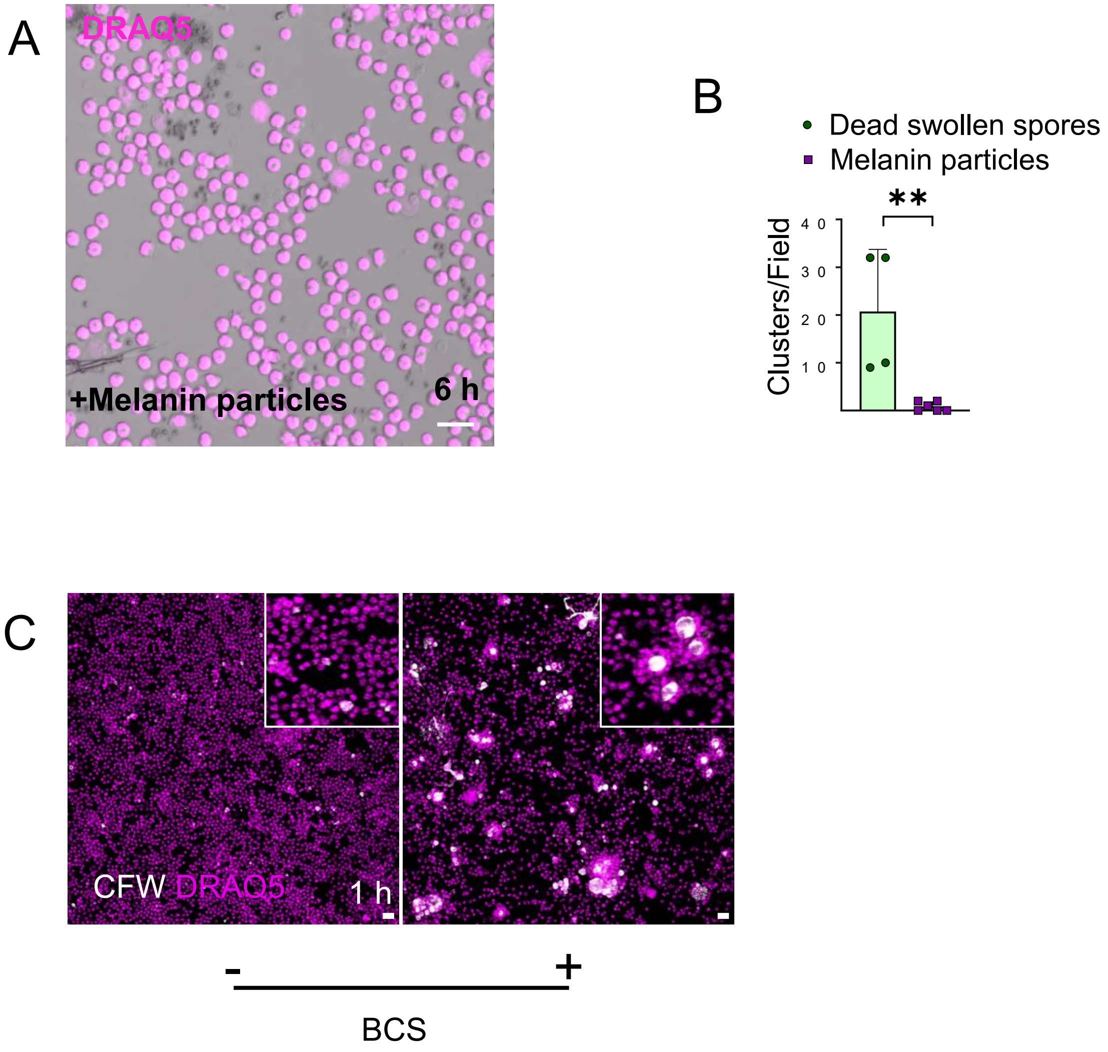

Supplementary Figure 5

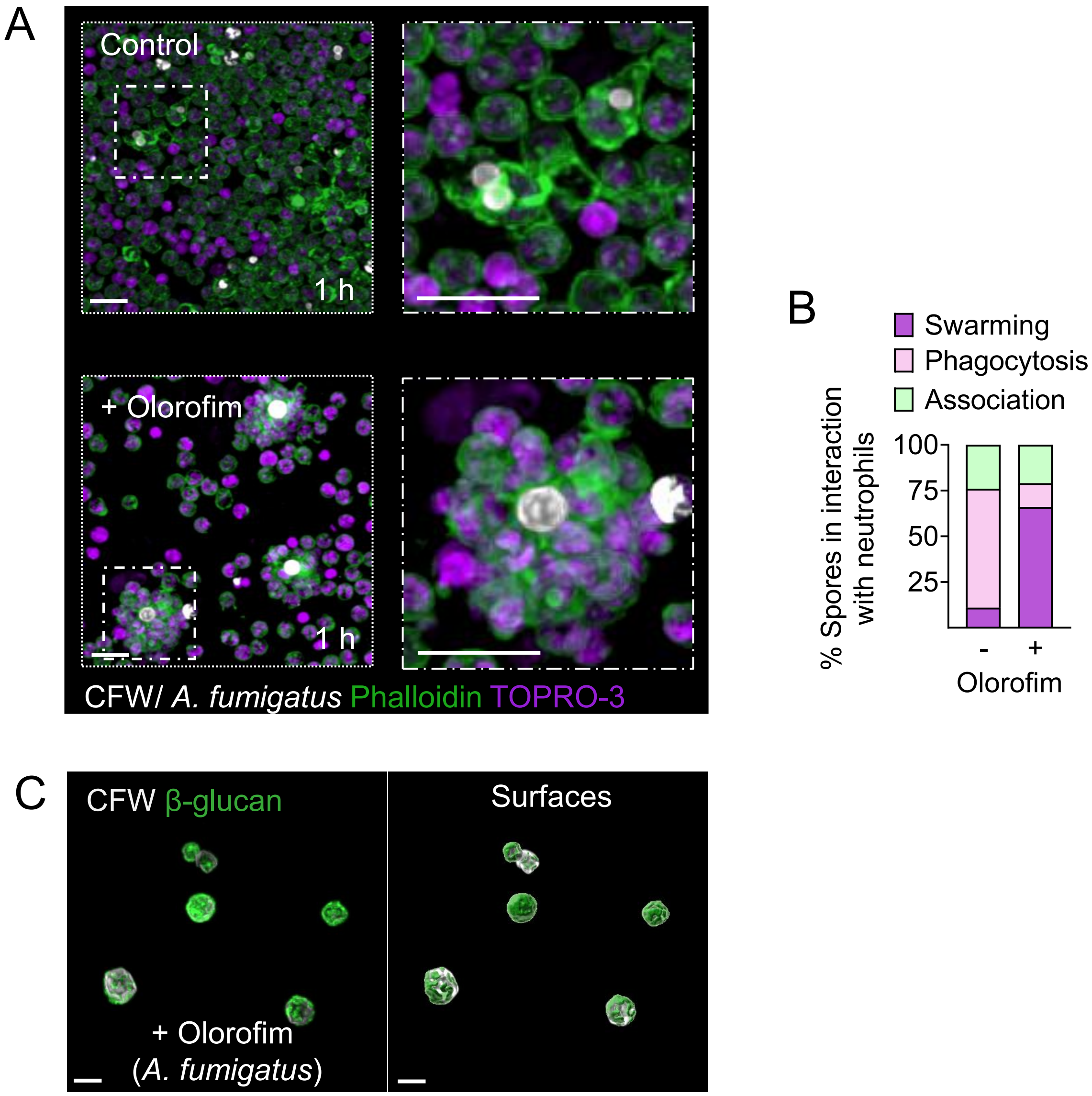

Supplementary Figure 6

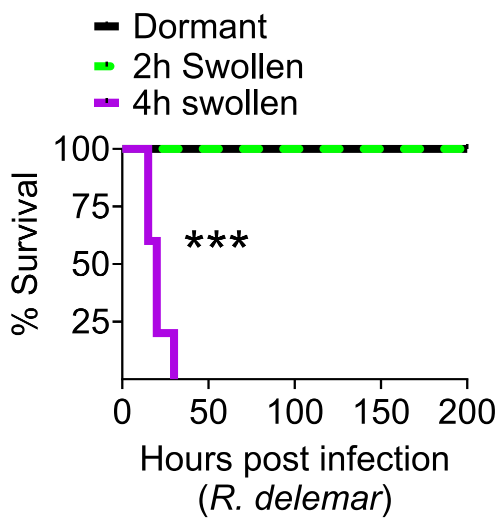

Supplementary Figure 7

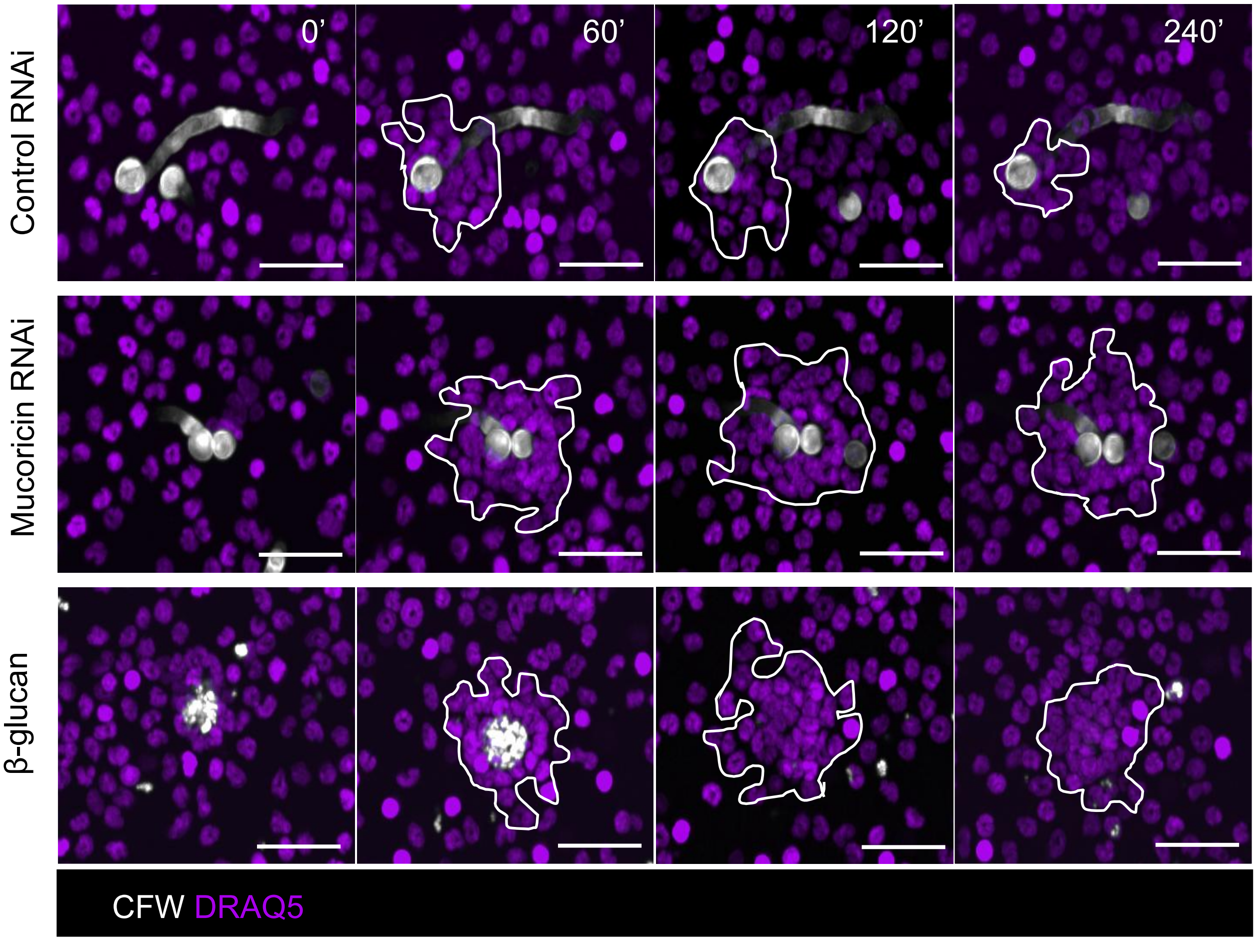

Supplementary Figure 8

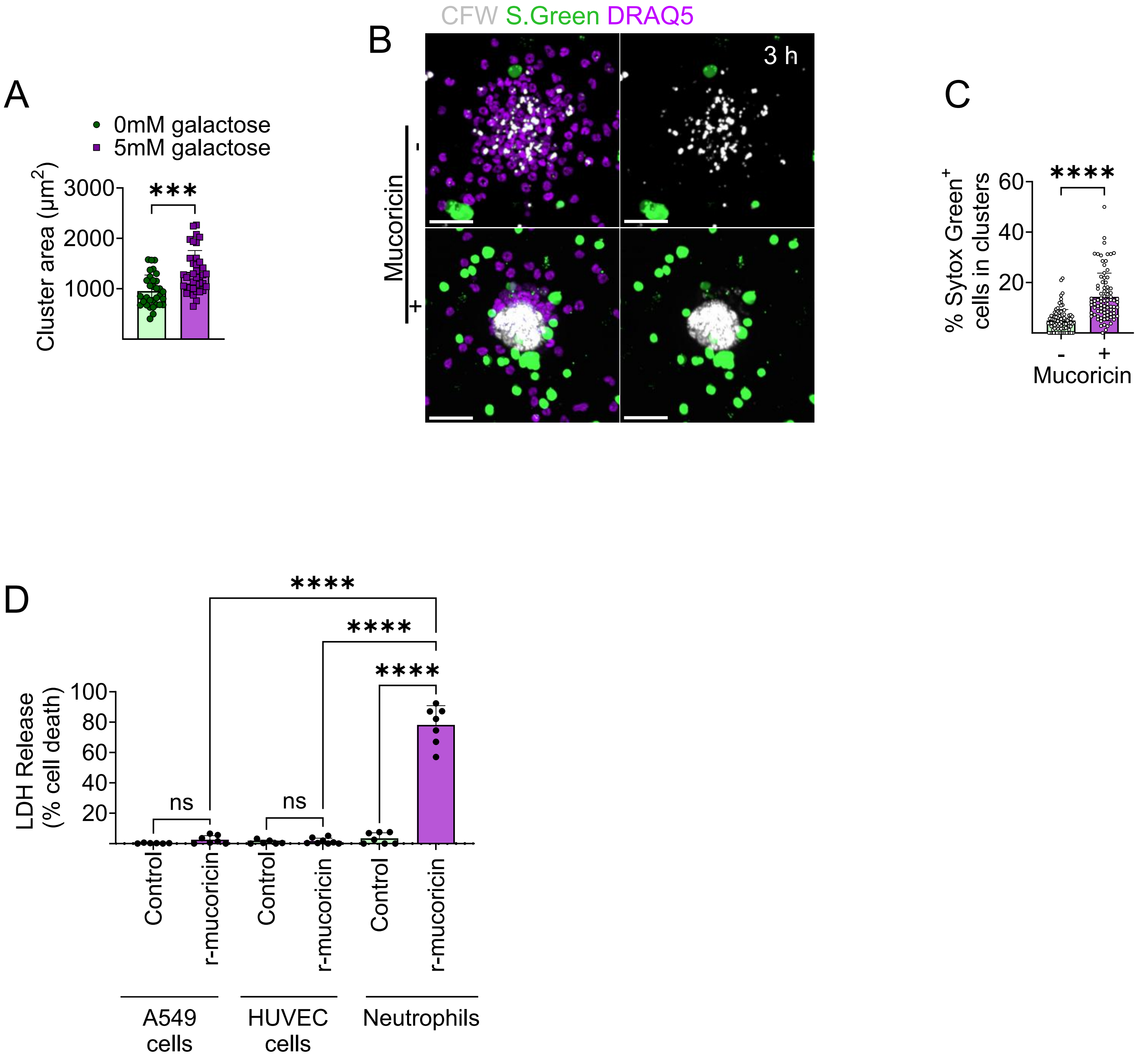

Supplementary Figure 9

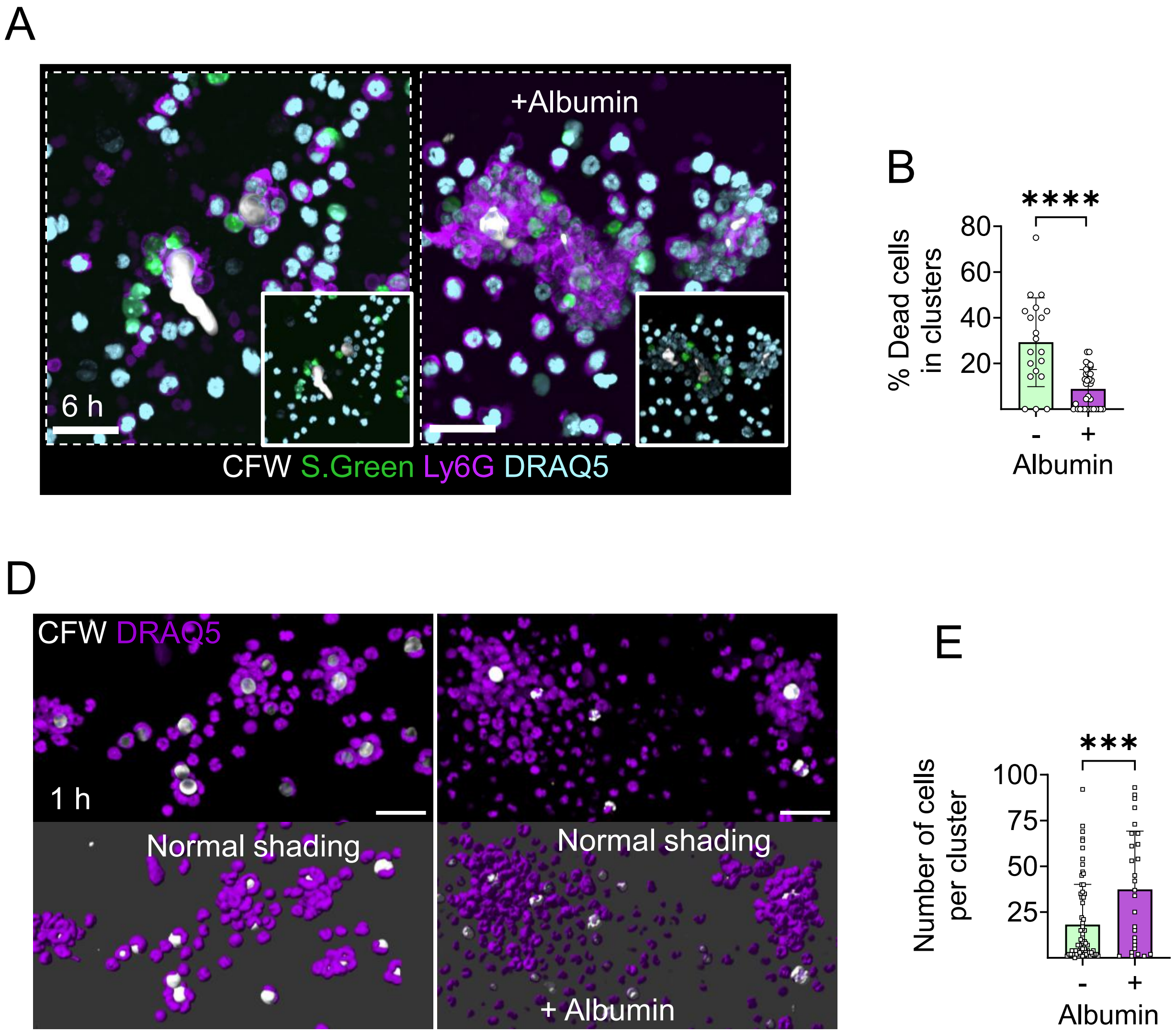

Supplement: Supplement 1 — Figure S1, related to Fig. 1 Ex vivo murine neutrophil swarming against towards Mucorales ex vivo. (A) Data on quantification of the number of spores engaged in different neutrophil BAL cell responses in the lungs of immunocompetent mice infected with either R. delemar at 24 h. (B) Representative images of murine bone-marrow neutrophils infected either with dormant R. delemar spores or A. fumigatus conidia, 4 hours post-infection. Scale bar, 10 μm. (C) Representative images of murine bone-marrow neutrophils infected either with dormant C. bertholletiae or M. circinelloides spores, at 4 h of infection. Scale bar, 30 μm. (D) Data on the quantification of different neutrophil responses in murine bone-marrow neutrophils infected either with C. bertholletiae or M. circinelloides. Figure S2, related to Fig. 1. Assessment of in vivo killing of R. delemar in the lung. (A) Schematic illustration of the protocol for assessment of in vivo killing of intra- and extracellular fungal spores. (Β) Percentage of killed R. delemar spores 3-, 6-, 12- and 24 h post infection of immunocompetent C57BL/6 (B6) mice with dormant fungal spores. The killing of intracellular spores begins earlier than the killing of the extracellular ones −3 and 6 h respectively, since there are many resident macrophages that phagocytose the spores, while neutrophils infiltrate the lung approximately 3 hours post infection. (C) Mice were sacrificed at the indicated time points, lungs were homogenized, and fungal loads were assessed by CFU plating. ***P = 0.005, ****P < 0.0001 One-way ANOVA. (D) Mean fungal area within macrophages in BAL of gp91phox +/+ mice and gp91phox −/− mice, infected with dormant R. delemar spores (n= 2/group). P=0.7094, Unpaired t-test. Figure S3, related to Figure 2. Human neutrophils swarm towards swollen R.delemar ex vivo. (A) Representative image of human neutrophils challenged with swollen R. delemar spores treated with PFA. Scale, 30 μm. (B) Data on quantification of the number of [file media-1.pdf]
